# Risk Factors for Delayed Diagnosis of Scabies in Hospitalized Patients From Long-Term Care Facilities

**DOI:** 10.4021/jocmr520w

**Published:** 2011-04-04

**Authors:** Chorng-Jang Lay, Chun-Lung Wang, Hui-Ying Chuang, Ya-Lan Chen, Hsiang-Ling Chen, Shu-Juan Tsai, Chen-Chi Tsai

**Affiliations:** a Division of Infectious Disease, Department of Medicine, Buddhist Dalin Tzu Chi General Hospital, Chiayi, Taiwan; b Infection Control Committee, Buddhist Dalin Tzu Chi General Hospital, Chiayi, Taiwan; c School of Medicine, Tzu Chi University, Hualien, Taiwan

## Abstract

**Background:**

Delayed diagnosis of scabies can cause an institutional outbreak, which causes considerably economic burden to control. This study was to find the risk factors for delayed diagnosis of scabies in hospitalized patients from long-term care facilities.

**Methods:**

We conducted a retrospective analysis of the hospitalized patients from long-term care facilities, diagnosed to have scabies between January 2006 and December 2008. A stepwise logistic regression analysis was performed to determine the risk factors for delayed diagnosis of scabies.

**Results:**

A total of 706 episodes with scabies were identified retrospectively in 399 hospitalized patients from long-term care facilities. Of these, 44 episodes were considered as delayed diagnosis of scabies. These patients were more associated with chronic usage of steroid (73% vs. 10%, P < 0.001) and had longer duration of hospitalization than the others (30 vs. 13 days, P < 0.001). After logistic regression, steroid therapy was the risk factor of delayed diagnosis of scabies (odds ratio: 23.493).

**Conclusions:**

In the patients from long-term care facilities, clinical physicians should pay more attention to those with chronic usage of steroid to avoid delayed diagnosis of scabies.

**Keywords:**

Scabies; Delayed diagnosis; Risk factor; Long-term care facility

## Introduction

Scabies, caused by *Sarcoptes scabiei var hominis* is a polymorphic disease that can cause protean cutaneous manifestations [1]. It distributes worldwide and affects 300 million individuals annually, encompassing all age groups and social classes [2]. With aging of population in recent decades, demand of long-term care facilities increased in industrialized countries. Crowded environment and centralized management in long-term care facilities accelerate the spreading of scabies and cause outbreaks [3]. When the patients with scabies are admitted to acute care facilities, delayed diagnosis causes institutional scabies outbreaks. Control of these outbreaks is associated with a considerable working and economic burden including ward closures, patient’s treatment and environmental disinfection procedures, laundry and extra staffing [4-6]. In addition, institutional scabies outbreaks may lead to adverse public perception of the establishment and panic among the residents’ family member, what is more, they cause a media frenzy and elicit fear of infection in medical personnel [7].

Scabies were found in 3.3% of the total population in long-term care facilities in Taiwan [8]. This patient group is the major population diagnosed to have scabies in hospitals. An epidemiological survey also showed that hospitals that possessed acute care and long-term care wards indicated higher prevalence of scabies [4]. Even though delayed diagnosis of scabies is the most important factor for outbreaks, however, there are no clinical studies to find the risk factors for delayed diagnosis of scabies in this patient group. Therefore, we conducted a retrospective study to find the risk factors for delayed diagnosis of scabies in hospitalized patients from long-term care facilities.

## Methods

### Study population

The hospitalized patients from long-term care facilities, diagnosed with scabies at Buddhist Tzu Chi Dalin General Hospital between January 2006 and December 2008 were enrolled in this study. This hospital was an 890-bed regional hospital with special ward for the patients from long-term care facilities. Their medical records were reviewed and the data including demographic characteristics, medical history, underlying diseases, medications, and laboratory data were collected for analysis.

### Definition of terms

A patient was considered to have scabies if any of the following was noted during his hospital stay: 1) clinical diagnosis according to the opinion of the dermatologist, 2) finding of scabies or its eggs in skin furfur under the microscope, 3) clinical diagnosis according their physicians and improvement of skin lesions under the therapy for scabies. The diagnosis was considered to be delayed if a patient was diagnosed with scabies 48 hours after admission. Corticosteroid treatment was defined as usage of a dose equivalent to at least 10 mg prednisolone per day for more than 14 days within 4 weeks before diagnosis of scabies [9]. Renal function impairment was defined as less than 50 ml/min of estimated creatinine clearance, calculated according to the laboratory data at admission. The diagnosis of cirrhosis was made on the basis of usual clinical, biological and endoscopic signs of liver disease. Chronic hypoxia (PaO_2_ < 60 mmHg), or carbon dioxide retention (PaCO_2_ > 55 mmHg) was considered as chronic respiratory failure. A patient was diagnosed with congestive heart failure by their physical condition such as pulmonary congestion, or peripheral edema, electrocardiography, chest film, and echocardiography by their clinical physicians. Leukocytosis was defined as more than 12 x 10^3^/μL in blood, and eosinophilia as more than 500/μL eosinophils in blood. Anemia was defined as less than 10 g/dL hemoglobulin in blood. Serum sodium ≤ 130 mmol/L was considered as hyponatremia, serum potassium ≤ 3.5 mmol/L as hypokalemia, and serum albumin ≤ 3g/dL as hypoalbuminemia. Chronically impairment of serum glutamic oxaloacetic transaminase was considered as liver function impairment. Bacteremia without apparent source was considered as primary bacteremia.

### Statistical analysis

SPSS 11.5 for MS Windows (SPSS, Chicago, IL, USA) software was used for the statistical analysis. Kolmogorov-Smimov test was used to test if the continuous data distributed normally. One-way ANOVA test was used for the continuous data not distributing normally and unpaired Student’s t test for the continuous data distributing normally. Pearson’s chi-squared or Fisher’s exact 2-tailed test was used to examine nominal data. Univariate analyses were used to identify the risk factors associated with delayed diagnosis of scabies. The independent predictors of delayed diagnosis were identified by stepwise logistic regression of multivariate analysis. A value of P < 0.05 was considered as statistically significant.

## Results

**Table 1:** Clinical Characteristics of Hospitalized Patients With Scabies From Long-Term Care Facilities

Age (years), mean ± SD	80 ± 8.8
Gender (male : female)	347 : 359
Duration from admission to diagnosis of scabies (days), mean (range)	1 (0 - 12)
Duration of hospitalization (days), mean (range)	14 (1 - 388)
Underlying disease	
Chronic respiratory failure, n (%)	288 (41)
Congestive heart failure, n (%)	96 (14)
End stage renal disease, n (%)	35 (5)
Scabies infection before, n (%)	360 (51)
Cirrhosis, n (%)	20 (3)
Pressure sore, n (%)	93 (13)
Malignancy, n (%)	45 (6)
Diabetes mellitus, n (%)	293 (42)
Hypertension, n (%)	567 (80)
Bedridden status, n (%)	610 (86)
Steroid therapy, n (%)	97 (14)
Anti-parkinsonism agent therapy, n (%)	103 (15)
Anti-seizure therapy, n (%)	186 (26)
Bacteremia during hospitalization, n (%)	225 (32)
Laboratory data	
White leukocyte (10^3^/μL), mean ± SD	13.3 ± 7.4
Eosinophil count (/μL), mean ± SD	410 (0 - 43025)
Hemoglobulin (g/dL), mean ± SD	10.6 ± 2.0
Serum sodium (mmol/L), mean ± SD	130.4 ± 9.1
Serum potassium (mmol/L), mean ± SD	4.1 ± 0.9
Serum creatinine (mg/dL), mean ± SD	1.3 ± 1.2
Serum urea nitrogen (mg/dL), mean ± SD	26.4 ± 22.5
Serum albumin (g/dL), mean ± SD	2.8 ± 0.7
Serum glutamic oxaloacetic transaminase (IU/L), mean (range)	35.6 (6 - 1375)

SD = standard deviation

A total of 706 episodes were identified retrospectively in 399 hospitalized patients from long-term care facilities. Their demographic data and clinical characteristics were summarized in Table 1. The mean age was 80 years (range, 33 to 98 years) and the overall duration of hospitalization was 14 days (range, 1 to 388 days). Mean duration from admission to diagnosis of scabies was 1 day (range, 0 to 12 days). Of 706 episodes, the patients were bedridden in 86% episodes, and had hypertension in 80% episodes. Forty-one percent of total episodes had chronic respiratory failure, 42% had diabetes mellitus, and 15% had ever had infected scabies. The patients were admitted due to bacteremia in the 225 episodes. Laboratory data within the first week of hospitalization in the 706 episodes was listed in Table 1.

**Table 2:** Univariate Analysis of Risk Factors for Delayed Diagnosis of Scabies in Hospitalized Patients From Long-Term Care Facilities

Variable	Delayed diagnosis (n = 44)	Early diagnosis (n = 662)	P value
Age (years), mean ± SD	78 ± 7.5	80 ± 8.9	0.223
Duration of hospitalization, days (range)	30 (6 - 388)	13 (1 - 221)	< 0.001*
Male, n (%)	27 (61)	320 (48)	0.129
Underlying disease			
Chronic respiratory failure, n (%)	18 (41)	270 (41)	1.000
Congestive heart failure, n (%)	4 (9)	92 (14)	0.501
End stage renal disease, n (%)	4 (9)	31 (5)	0.266
Scabies infection before, n (%)	19 (43)	341 (52)	0.360
Cirrhosis, n (%)	0 (0)	20 (3)	0.629
Pressure sore, n (%)	8 (18)	85 (13)	0.433
Malignancy, n (%)	5 (11)	40 (6)	0.190
Diabetes mellitus, n (%)	20 (45)	273 (41)	0.695
Hypertension, n (%)	32 (73)	535 (81)	0.267
Bedridden status, n (%)	41 (93)	569 (86)	0.259
Steroid therapy, n (%)	32 (73)	65 (10)	< 0.001*
Anti-parkinsonism therapy, n (%)	4 (9)	99 (15)	0.397
Anti-seizure therapy, n (%)	13 (30)	173 (26)	0.748
Bacteremia, n (%)	15 (34)	210 (32)	0.873
Laboratory data			
Leukocytosis, n (%)	24 (55)	378 (59)	0.862
Eosinophilia, n (%)	12 (27)	116 (18)	0.104
Anemia, n (%)	22 (50)	243 (37)	0.109
Hyponatremia, n (%)	28 (64)	450 (68)	0.647
Hypokalemia, n (%)	11 (25)	156 (24)	0.982
Renal function impairment, n (%)	10 (23)	145 (22)	1.000
Hypoalbuminemia, n (%)	32 (73)	454 (69)	0.684
Liver function impairment, n (%)	12 (27)	181 (27)	1.000

SD = standard deviation *: P < 0.05

Of 706 episodes, 44 episodes were considered as delayed diagnosis of scabies. Risk factors of delayed diagnosis of scabies in univariate analyses were listed in Table 2. The patients with delayed diagnosis of scabies had longer duration of hospitalization than the others (30 vs. 13 days, P < 0.001). The ratio of the patients receiving steroid therapy chronically before admission was higher in the group of delayed diagnosis than in the other group (73% vs. 10%, P < 0.001). Other variables were not associated statistically with delayed diagnosis of scabies. The result of logistic regression analysis was listed in Table 3. Steroid therapy was the most important risk factor of delayed diagnosis of scabies (odds ratio: 23.493 and 95% confidence interval: 11.354 - 48.612).

**Table 3:** Multivariate Logistic Regression Analysis of Risk Factors Associated With Delayed Diagnosis of Scabies in the Hospitalized Patients From Long-Term Care Facilities

Variable	Odds ratio	95% confidence interval	P value
Steroid therapy	23.493	11.354 - 48.612	< 0.001

Bacteremia was noted during hospitalization in the 225 episodes. Of these, primary bacteremia was noted in the 152 episodes. The most common pathogen for primary bacteremia was coagulase-negative *Staphylococcus* (72%), followed by *Staphylococcus aureus (12%)*, and un-identified gram-positive *bacilli* (11%). The distribution of pathogens of primary bacteremia was listed in Table 4.

**Table 4:** Distribution of Pathogens of Primary Bacteremia in Hospitalized Patients With Scabies From Long-Term Care Facilities

Pathogens	No. of episodes
Coagulase-negative *Staphylococci*	110
*Staphylococcus aureus*	18
Unidentified gram-positive *bacilli*	16
*Streptococci*	3
*Bacillus* spp.	2
*Enterococci*	2
*Acinetobacter* spp.	1

## Discussion

In a review of 20 hospital outbreaks of scabies, 16 of 19 index cases were considered to have crusted scabies, 16 of 19 index cases were considered to have crusted scabies or a variant [7]. Another review analyzed 19 outbreaks; all but one of the index patients had crusted scabies [10]. Patients with crusted scabies serve as a reservoir for the mite and their atypical presentation is the major cause for delayed diagnosis of scabies in these outbreaks. Several factors had been found for atypical clinical features of scabies, including HIV infection, human-T cell leukemia/lymphoma virus type 1 infection, organ transplant, elderly or debilitated individuals as well as long-term immunosuppressive medications [10]. Of these factors, our study proved chronic usage of steroid was the most important factor for delayed diagnosis of scabies in hospitalized patients from long-term care facilities. To date, there is no general policy for early and systemic detection of scabies for newcomers to a healthcare setting. However, according to the result of our study, scabies should be considered as a differential diagnosis for any atypical skin lesion on patients with chronic usage of steroid from long-term care facilities in order to avoid delayed diagnosis.

Scabies mites reside in the lower stratum corneum of the epidermis near the stratum corneum/stratum lucidum interface [11]. The intercellular fluid from lower skin zones seeps into the burrows and provides a medium for soluble antigens from the mite’s body and feces to diffuse into the dermis and stimulate an inflammatory and immune response. Clinical symptoms of scabies result from a delayed host immune response to these soluble antigens [12]. Besides alleviating itching sensation, steroid could alleviate local inflammation which progresses their lesions to be camouflaged and exacerbated [13-15]. Instead of classical manifestation of scabies, this kind of patients presents as scaly, crusted papules, urticarial papules, or persistent nodules lesions [16, 17]. Orkin had ever described seven manifestations of scabies, one of which called scabies incognito as a result of modified appearance of the lesions due to use of steroids [18]. Due to variable presentations of scabies incognito, they can be easily ignored only under naked eye even by the experienced dermatologist.

Due to imprecise diagnosis of atypical skin lesions of scabies only under naked eye, skin lesions of patients with chronic usage of steroid from long-term care facilities should be microscopically confirmed. Traditionally, an oil preparation of a skin scarping or material retrieved from underneath the nails enables visualization of mites, or eggs. However, correct performance of scarping techniques requires some skill and learning. Epiluminescence microscopy is an in vivo technique with high sensitivity that allows rapid diagnosis without patient inconvenience [19]. In addition, a tape stripping method seems easier and useful to detect mites in bedridden elderly patients [20].

In previous studies, scabies is frequently associated with superinfection caused by *Streptoccus pyogenes* or *Staphylococcus aureus*, which may be associated with increased morbidity and occasionally fatal outcome [21-23]. In some reports, *S. aureus* is the most prevalent aerobic bacterium, recovered from local scabies lesions. Staphylococcal superinfection may complicate crusted scabies increasing morbidity and even mortality [10]. The same phenomenon was noted in our study, in which high ratio of patients with scabies had primary bacteremia and almost all pathogens belonged to normal skin flora. Delayed diagnosis may give more change for bacterial superinfection in patients with scabies. This may explain why longer hospitalization happened in patients with delayed diagnosis of scabies.

In conclusion, scabies is especially common among elderly patients living in long-term care facilities. Avoiding delayed diagnosis is most important to prevent scabies from spreading in the hospitals. According the result of our study, clinical physicians should pay more attention to any skin lesion in the patients receiving steroid chronically from long-term care facilities for scabies. Their skin lesions should be microscopically confirmed immediately after admission to avoid delayed diagnosis of scabies.
